# Development and Application of Infrared Thermography Non-Destructive Testing Techniques

**DOI:** 10.3390/s20143851

**Published:** 2020-07-10

**Authors:** Zhi Qu, Peng Jiang, Weixu Zhang

**Affiliations:** 1 State Key Laboratory for Strength and Vibration of Mechanical Structures, Xi’an Jiaotong University, Xi’an 710049, Shanxi, China; quzhcn001@stu.xjtu.edu.cn (Z.Q.); jiangpeng219@mail.xjtu.edu.cn (P.J.); 2 School of Engineering and Applied Science, Harvard University, Cambridge, MA 02138, USA

**Keywords:** non-destructive evaluation, thermal wave, thermal excitation, infrared image, defects

## Abstract

Effective testing of defects in various materials is an important guarantee to ensure its safety performance. Compared with traditional non-destructive testing (NDT) methods, infrared thermography is a new NDT technique which has developed rapidly in recent years. Its core technologies include thermal excitation and infrared image processing. In this paper, several main infrared thermography nondestructive testing techniques are reviewed. Through the analysis and comparison of the detection principle, technical characteristics and data processing methods of these testing methods, the development of the infrared thermography nondestructive testing technique is presented. Moreover, the application and development trend are summarized.

## 1. Introduction

Non-destructive testing (NDT) refers to the technology that uses acousto-optoelectronics and electromagnetism to detect internal defects in materials or structures without damaging their effectiveness and reliability; it is then used to understand and evaluate the properties, condition, and quality of the tested objects [1,2,3,4,5,6,7,8,9,10,11]. The traditional non-destructive testing methods primarily involve acoustic emission, penetration testing, eddy current testing, ultrasonic testing, ray testing and other technologies [12,13,14,15,16,17,18,19,20]. With the increasing demand for non-destructive testing in aerospace and other high-tech industries, greater significance is being placed on developing a new non-destructive testing technology. Therefore, to address the shortcomings of traditional testing methods, a new technique-infrared thermography testing has been rapidly developing in recent years. As a new NDT method, infrared thermography has the advantages of being able to inspect large areas and delivering intuitive detection results quickly and easily [4]. The comparison with traditional non-destructive testing is shown in Table 1. This technique was first introduced by in 1980s. Since then, relevant research has been quickly progressing all over the world. From various countries, scholars such as Tuli, Maldague, Vavilov, Almond, and Busse [21,22,23,24,25,26] have laid a solid theoretical foundation. In addition, Schroeder [27], Shepard [28], Mulaveesala [29], Favro [30], etc., as well as many research institutes, have further contributed to research on this technique [31,32,33,34,35,36,37,38,39,40,41,42,43,44,45,46,47,48,49,50,51]. Because of these global efforts, infrared thermal wave imaging non-destructive testing has been widely accepted.

In this paper, firstly, the law of thermal wave propagation in objects is introduced. Then, the infrared image processing technologies are summarized. Finally, their applications are illustrated, and the development trend is prospected.

## 2. Principle and Classification

### 2.1. Theoretical Basis

Heat flow is associated with measurable temperature scales, but it cannot be measured directly. Therefore, once the temperature distribution *T(r,t)* in an object is determined, the heat flow can be calculated using a law connecting heat flow with temperature, or Fourier’s law [4]

(1) q(r,t)=−k∇T(r,t)

where *q(r,t)* represents the heat flux per unit time on the unit isothermal surface in the direction of temperature reduction, *k* is the thermal conductivity of the material, and ∇*T(r,t)* is the temperature gradient.

Fourier’s law shows the relationship between heat flux and the temperature gradient, and it is useful for both steady and unsteady fields [5]. Then, the differential equation of heat conduction used to describe the internal relationship of the temperature field in the time–space domain is

(2) $$\nabla^{2}T(r,t)+\frac{q_{v}}{k}=\frac{1}{\alpha }\frac{\partial T(r,t)}{\partial t}$$

where α=k/ρc is the thermal diffusivity, and *q_v_* denotes the term of the heat source. From this, the theoretical model of infrared thermal imaging non-destructive testing can be analyzed by combining this equation with the boundary conditions.

In terms of radiation, the total radiation intensity of a gray body is equal to the total radiation intensity of a blackbody, multiplied by the emission coefficient of the gray body; that is, the radiation of a gray body satisfies Stephen–Boltzmann law [6,7]

(3) $$W=\varepsilon \sigma T^{4}$$

where *ε* is the emission coefficient of the gray body, *σ* is the Stephen–Boltzmann constant, *W* and *T* are the radiation intensity and absolute temperature of the object, respectively.

**Figure 1:**
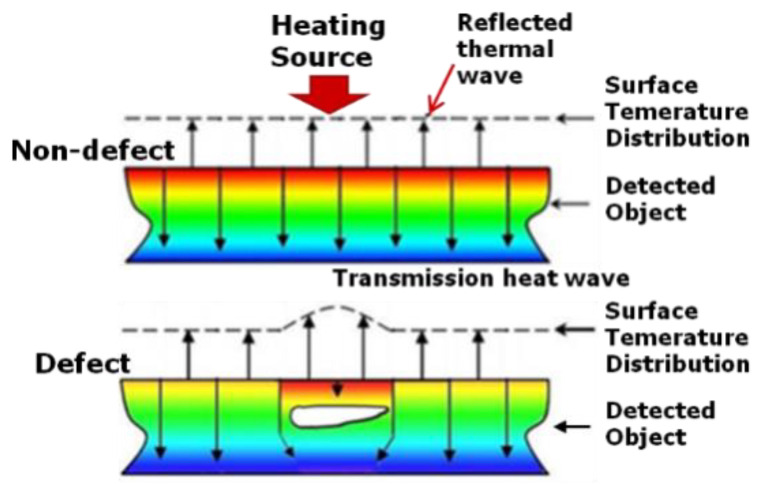
Principle of infrared thermal wave imaging detection.

Infrared thermography testing uses the corresponding relationship between thermal radiation and temperature. With different forms of active thermal excitation, the heteromorphic structure of the object can be represented by the difference of the surface temperature distribution, and then the defect can be accurately located and identified. As shown in Figure 1, When the thermal signal is applied to the surface of the object, if the material is uniform and has no defects in its propagation direction, the thermal wave will propagate smoothly in the body. Finally, the thermal response signal accumulated on the surface is uniformly distributed, that is, the temperature distribution on the surface of the specimen is the same, and there is no abnormality. If there are defects in the specimen, the reflection will occur when the heat wave propagates to the defect, resulting in the sudden change of surface temperature distribution.

At present, the research and application of infrared thermography non-destructive testing technology mainly focus on the following aspects: (1) Mechanism of interaction between thermal wave and defective materials. The process of thermal wave imaging includes the propagation, reflection and scattering of transient thermal wave in the detected object, that is, the mechanism and representation of the interaction between the transient thermal wave generated by thermal excitation and the internal structure and interface of the detected object. The current theoretical research is mainly focused on the regular defects of isotropic plate structure, and the theoretical solution of the surface temperature field after thermal excitation is derived, so as to comprehensively understand and regularly summarize the detection methods. (2) Infrared thermal image sequence processing. Due to the interference of many factors, the original thermal image contains much noise. Especially for the defects with large depth or small size, due to the small temperature difference, amplitude difference and phase difference, it is easy for them to be submerged in the noise. The main infrared image processing and analysis methods include: infrared image nonuniformity correction, enhancement, noise reduction segmentation, and more. These methods generally deal with a single image, which can improve the signal-to-noise ratio and defect display effect to a certain extent. (3) Quantitative identification of defects. This problem belongs to the inverse problem of heat conduction, which is mainly through the extraction and processing of temperature, amplitude and phase in the non-destructive and abnormal areas of the thermal image. Set the threshold and compare the test results, and finally quantitatively detect the type, size and depth of defects [52,53,54,55,56,57,58,59,60,61,62,63,64,65,66,67,68].

### 2.2. Classification of Infrared Thermal Wave Testing

This paper reviews several main methods of infrared thermography non-destructive testing, focusing on the theoretical solution, technical characteristics and limitations of each method. Each method has its own applicable conditions. According to different material types and defect forms, appropriate detection methods should be selected.

#### 2.2.1. Infrared Pulsed Thermography Testing

**Figure 2:**
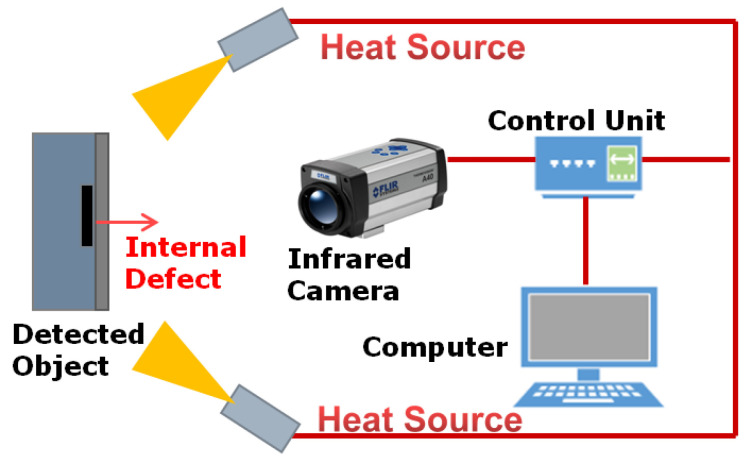
Process of infrared thermography non-destructive testing.

Infrared pulsed thermography testing is one of the most studied and well-developed infrared non-destructive testing technologies in the world [19,20,23,24,45,46,47,48,49,50,51]. This method uses a high-energy pulsed flashing lamp as an excitation heat source, as shown in Figure 2. If defects or damage in the test object exist, the discontinuous structure of the object will lead to a significant abnormality in the surface temperature. If defects or damage existed in the test object, the discontinuous structure of the object will lead to a significant abnormality in the surface temperature. This technology has the characteristics of fast detection speed, large detection area, and convenience for online detection. It can detect the defects such as debonding, crack, rust, fatigue damage in metal, non-metal and composite materials. It is increasingly becoming an important means to ensure the product and safe operation, and has a broad development prospect. At present, the most used pulse excitation sources mainly include flash lamp, laser, infrared lamp hot air, and more. The comparison of characteristics and application is shown in Table 2. Among these exciting heat sources, the high-power flash lamp is the most widely researched and applied pulse loading mode in the world. 

When a pulsed heat source is used to irradiate the test object, the surface of the object satisfies the one-dimensional heat conduction equation:

(4) $$\frac{\partial^{2}T\left(x,t\right)}{\partial x^{2}}-\frac{\rho c}{k}\frac{\partial T\left(x,t\right)}{\partial t}=-\frac{q_{0}\delta \left(t\right)}{k}$$

where $q_{0}$ is the heating intensity, which refers to the power of heat source, δ(t) is the unit pulse function, *x* is the direction of heat flow injection and propagation, *k* is heat transfer coefficient, *ρ* is density, *c* is specific heat, *T* is temperature, and *t* is time. Assuming that the object is a semi-infinite space, the temperature of the specimen is then obtained by solving the equation

(5) $$T_{n}\left(x,t\right)=\frac{q_{0}}{\sqrt{\pi \rho ckt}}\exp\left(-\frac{x^{2}}{4\alpha t}\right)$$

where α is thermal diffusion coefficient. When the thermal wave propagates to the defect at a depth *d* from the surface, it will be stopped and reflected. Then, the corresponding surface temperature of the defect area is found by

(6) $$T_{d}\left(0,t\right)=\frac{q_{0}}{\sqrt{\pi \rho ckt}}\left[1+2\exp\left(-\frac{d^{2}}{\alpha t}\right)\right]$$

Finally, the surface temperature difference is obtained using the equation

(7) $$\Delta T=T_{d}\left(0,t\right)-T_{n}\left(0,t\right)=\frac{2q_{0}}{\sqrt{\pi \rho ckt}}\exp\left(-\frac{d^{2}}{\alpha t}\right)$$

Therefore, after pulse heating, the defect depth can be determined based on time; this corresponds to the peak temperature difference in the equation

(8) $$t_{\max}=\frac{2d^{2}}{\alpha }$$

Although the pulse infrared thermography detection method is simple and practical, it also has shortcomings. For example, this technology works well for defect detection in flat panel components but has difficulty with complex structural components. Moreover, it is limited by the thicknesses of the object to be inspected; therefore, if the test object thickness is large, infrared thermography will have difficulty detecting the defect. In addition, the requirement for uniformity of the pulsed heat source is usually very high.

#### 2.2.2. Infrared Lock-in Thermography Testing

Infrared lock-in thermography uses periodic modulated heat source to heat the tested object periodically. If there is a defect in the object, the defect will have a periodic effect on the surface temperature distribution above it, even at fairly low peaks. Therefore, there will be a phase difference between the defective place and the non-defective place. The advantage of phase angle is that it is less sensitive to local changes in light and/or surface emissivity, because the depth resolution of its single frequency excitation test is fixed (i.e., fixed thermal wavelength). The infrared thermal imager collects the surface temperature distribution of the object. There are noise signals, DC signals and other interference signals in the collected heat map sequence. The purpose of lock-in is to separate the weak useful signals from many interference signals. The thermal excitation sources used in the phase-locked method include halogen lamp/infrared lamp, laser, ultrasonic, electromagnetic, and more [51,52,53,54,55,56,57,58,59,60,61,62,63,64].

Under the excitation of modulated heat source, the response of a one-dimensional heat wave in an isotropic and homogeneous medium is

(9) $$T\left(x,t\right)=T_{0}e^{-x/\mu_{0}}\cos\left(\frac{2\pi x}{\lambda }-2\pi ft\right)$$

where *f* is the frequency, λ is the wave length, and $\mu_{0}=\sqrt{\alpha /\pi f}$ is the depth of thermal diffusion. According to the literature [60], the depth of the defect is proportional to $\mu_{0}$, specifically, it is approximately $1.5\mu_{0}\sim 2\mu_{0}$. *T*_0_ is the amplitude of the surface temperature.

Compared with infrared pulsed thermography, this technique has the following advantages: it is not affected by uneven heating; the phase diagram has nothing to do with the emissivity of the component’s surface; and the low heating temperature will not cause damage to the material’s surface. In addition, phase and amplitude detection comparison can be significantly improved, ultimately improving the detection and measurement of defects, by improving the accuracy of the thermal imager. One disadvantage of this method is that different modulation frequencies must be tried during the detection process. If the frequency is too low, the signal-to-noise ratio (SNR) of the thermal image is low, resulting in a much longer single experiment cycle. Conversely, if the frequency is too high, the penetration depth of the thermal wave is not deep enough [53,54,55,56,57,58,59].

The basic principle of the current lock-in thermography testing is based on the condition that the object under testing is under the action of sinusoidal temperature field. However, in practice, especially in the low-frequency case, the sinusoidal temperature field heat source will have waveform distortion due to the influence of the environment and so on, which will affect the phase discrimination of the collected surface temperature field signal. The heating method of square wave has a definite phase. The heat source of square wave is a periodic rectangular pulse signal, and the Fourier transform of its power *P(t)* is [58,59]:

(10) $$P\left(t\right)=\frac{A\tau }{T_{1}}+2\frac{A\tau }{T_{1}}\sum_{n=1}^{\infty }Sa\left(\frac{n\pi \tau }{T_{1}}\right)\cos n\omega_{1}t$$

where *A* is amplitude, *τ* is pulse width, and *T*_1_ is period time.

Its spectrum energy is mainly concentrated in the first zero point. The energy of high-order frequency component is low, which can be ignored in practical analysis. In addition, the non- fundamental component can be known from the orthogonality principle that it is zero after multiplying with the switch function and integrating in the whole lock-in period. Therefore, the acquisition time in the experiment is required to be an integral multiple of the lock-in period, so the DC component and the high-frequency component are suppressed after the signal processing, and the effect is the same as using a single frequency sinusoidal heat source. This method is relatively simple.

#### 2.2.3. Infrared Ultrasonic Thermography Testing

Infrared ultrasound thermography combines ultrasonic excitation technology with infrared thermal imaging technology. Using ultrasonic energy as the thermal excitation source, a 20–40 kHz ultrasonic wave is transmitted into the test object. If there is a defect in the object, the ultrasonic energy of the high-frequency vibration will cause friction heat at the defect interface. The thermal imager captures changes in the temperature of the test object’s surface, thus detecting the defect [53,61,62,63,64]. By using the defect location itself to generate heat, this method is less affected by background noise and obtains high contrast thermal images. However, in the process of thermal excitation, the source must transmit ultrasonic energy into the test object under certain pressure; this pressure could easily cause secondary damage to the object [65].

Friction is the main cause of local heat generation in the defect area when the defect interface is excited by the ultrasonic wave. Assuming that the thermoelastic and hysteresis effects can be neglected, the internal action of the ultrasonic wave is described by the following equations [53]:

(1) Ultrasonic wave propagation in sheet metal.
  The displacement partial differential control equation (Navier governing equation) is
  (11) $$(\lambda +\mu )\frac{\partial^{2}u_{i}}{\partial x_{i}^{2}}+\mu \frac{\partial^{2}u_{i}}{\partial x_{i}\partial x_{j}}+\rho f_{i}=\rho \frac{\partial^{2}u_{i}}{\partial t^{2}}$$
  where *u_i_* and *f_i_* are the position and volume force tensors, respectively, *μ* is the trimming modulus *λ* is the lame constant, and *ρ* is the component density.
(2) Ultrasonic vibration heat generation at defect contact interface.
  The heat flux *Q*(*t*) is calculated by the equation
  (12) $$Q(t)=[\mu_{d}+(\mu_{s}-\mu_{d})e^{-cv\left|\left(t\right)\right|}]F_{N}(t)v_{\tau }(t)$$
  where *v*(*t*) and $v_{\tau }(t)$ are the relative and tangential velocity of the interface contact point respectively, and *μ_s_* and *μ_d_* are the static and dynamic friction coefficients at the defect, respectively. *F_N_*(*t*) is the contact force, and *c* is the velocity coefficient for converting static friction into dynamic friction.
(3) Conduction of heat flow at the defect.
  The propagation of the heat flux *Q* caused by ultrasonic stimulation at the defect satisfies the heat conduction differential equation
  (13) $$k\frac{\partial^{2}T}{\partial x_{i}^{2}}+q=\rho c\frac{\partial T}{\partial t}$$
  where *k* and *c* are the thermal conductivity, density, and specific heat of object, respectively.

Equations (11) to (13) establish an analytical model of acoustic–mechanical–thermal energy coupling for ultrasonic excitation of a metal plate with contact interface defects. Under given initial and boundary conditions, these equations can be solved to obtain changes in the tested object’s surface temperature.

#### 2.2.4. Infrared Laser Scanning Thermography Testing

In the traditional infrared thermal imaging detection methods introduced above, the thermal wave only transmits in the depth direction; there is no heat conduction in the horizontal direction. Thus, the traditional method can only detect horizontal cracks but is not able to effectively detect vertical cracks. Therefore, for the detection of vertical cracks, infrared laser scanning thermal imaging has become the focus of research in recent years [69,70,71,72]. In 2011, Burrows et al. [73,74] detected surface cracks on stainless steel and pure aluminum components by scanning point laser; this method was based on the principle that temperature rises at surface cracks during scanning. In the same year, Li et al. inspected the surface cracks of austenitic stainless steel by using the point heat source formed by a laser point near the crack; this method was based on the thermal resistance of the surface cracks. In 2012, Zhang et al. [74] proposed the grating infrared thermal wave method, which retains the high efficiency of traditional heat propagation to detect both the horizontal and vertical cracks. In 2013, An et al. [75] used the thermal resistance phenomenon at the crack site combined with the post-processing method of lock-in temperature amplitude to detect fatigue cracks on metal components. In 2014, Liu et al. [76,77,78] used the multi-mode scanning laser thermography method for surface crack detection in thermal barrier coatings. 

(1) Point Source

After applying a point heat source at *t* = 0, the temperature gradient field in a test object is distributed as follows:

(14) $$T(x,y,z,t)=\int_{0}^{t}\int_{-\infty }^{+\infty }\int_{-\infty }^{+\infty }\int_{-\infty }^{+\infty }\frac{Q(x^{\prime },y^{\prime },z^{\prime },t^{\prime })}{C\rho {\left[4\pi a(t-t^{\prime })\right]}^{\frac{3}{2}}}\cdot e^{-\frac{{\left(x-x^{\prime }\right)}^{2}+{\left(y-y^{\prime }\right)}^{2}+{\left(z-z^{\prime }\right)}^{2}}{4a(t-t^{\prime })}}\cdot dt^{\prime }dx^{\prime }dy^{\prime }dz^{\prime }$$

where *Q* is the electric heat source instantaneous heat, *ρ* is density, *C* is specific heat capacity, and *α* is thermal diffusivity.

It is known that the common laser distribution is Gaussian. Therefore, if the central power density of a laser is *P*_0_, and the radius of the spot is *R*, then the power density distribution function is

(15) $$P(x,y)=\frac{2P_{0}}{\pi R^{2}}e^{-2\frac{{\left(x-x_{0}\right)}^{2}+{\left(y-y_{0}\right)}^{2}}{R^{2}}}$$

Assuming the absorption coefficient of the sample surface to light energy is 0, the temperature gradient distribution on the sample surface at *t* time can be obtained under the action velocity *v* along the *x*-axis direction of Gauss distribution laser scanning by

(16) $$T(x,y,z,t)=\int_{0}^{t}dt^{\prime }\frac{2\;\rho_{0}P_{0}\sqrt{\alpha }}{k\pi^{\frac{3}{2}}[8\alpha {\left(t-t^{\prime }\right)}^{\frac{3}{2}}+R^{2}\cdot {\left(t-t^{\prime }\right)}^{\frac{1}{2}}]}e^{-2\frac{{\left(x-x_{0}-vt^{\prime }\right)}^{2}+{\left(y-y_{0}\right)}^{2}}{8\alpha (t-t^{\prime })+R^{2}}-\frac{z^{2}}{4\alpha (t-t^{\prime })}}$$

(2) Line heat source

If we use the Gaussian distribution of a line heat source, we need to integrate the *y* direction by

(17) $$T(x,z,t)=\frac{2I}{2l\cdot 4\pi k}\int_{-l}^{l}d\lambda \int_{0}^{t}\sin(\omega \tau )\cdot \frac{e^{\frac{-{\left(x-\lambda -v\tau \right)}^{2}}{4\alpha (t-\tau )}}}{\left(t-\tau \right)}d\tau$$

Then, the surface temperature field can be obtained with

(18) $$\begin{array}{l} T(x,z,t)=\frac{I}{8L\cdot l\cdot \pi k}\cdot \int_{0}^{t}\sin(\omega \tau )\cdot \frac{\sqrt{\alpha \pi }}{\sqrt{t-\tau }}[erf(\frac{\tau v+l-x}{\sqrt{4\alpha (t-\tau )}}-erf(\frac{\tau v-l-x}{\sqrt{4\alpha (t-\tau )}})]d\tau \\ erf(x)=\frac{2}{\sqrt{\pi }}\cdot \int_{0}^{x}e^{-\eta^{2}}d\eta \end{array}$$

The rapid attenuation of thermal wave signals along the depth direction makes the defect-free area look like a semi-infinite space. Correspondingly, the defect area can be regarded as a finite thickness area. For the analysis of finite thickness heat conduction, the mirror heat source method is usually used. This method treats the adiabatic boundary as a mirror and the temperature distribution anywhere in the medium as the superposition of the real heat source and the mirror heat source effect. Considering the effect of each heat source, the temperature distribution of the test object with a defect thickness of *d* is

(19) $$T(x,z,t)=\frac{I}{8L\cdot l\cdot \pi k}\cdot \int_{0}^{t}\frac{\sqrt{\alpha \pi }}{\sqrt{t-\tau }}\cdot (1+2e^{\frac{-d^{2}}{\alpha (t-\tau )}})\cdot [erf(\frac{\tau v+l-x}{\sqrt{4\alpha (t-\tau )}}-erf(\frac{\tau v-l-x}{\sqrt{4\alpha (t-\tau )}})]d\tau$$

#### 2.2.5. Grating Infrared Thermal Wave Scanning Testing

Thermal excitation with high energy and short pulses as well as high frame rate infrared image acquisition are the two main technologies for infrared thermal wave imaging detection, especially for materials with high thermal conductivity and defects close to the surface. For the detection of thin films, especially materials with high thermal conductivity, the time of thermal excitation must be very short because of the fast change of thermal wave signals. Otherwise, the thermal excitation will not end when the echo of the thermal wave reaches the surface of the test object; this will affect the detection accuracy. The grating infrared thermal wave scanning method uses a sinusoidal linear grating to scan the test object surface rapidly, forming high-density, pulse-modulated thermal excitation. The advantage of this method is that at the same frequency, the conduction attenuation of thermal wave signals along the depth direction is faster, and the thermal diffusion depth is smaller [66].

**Figure 3:**
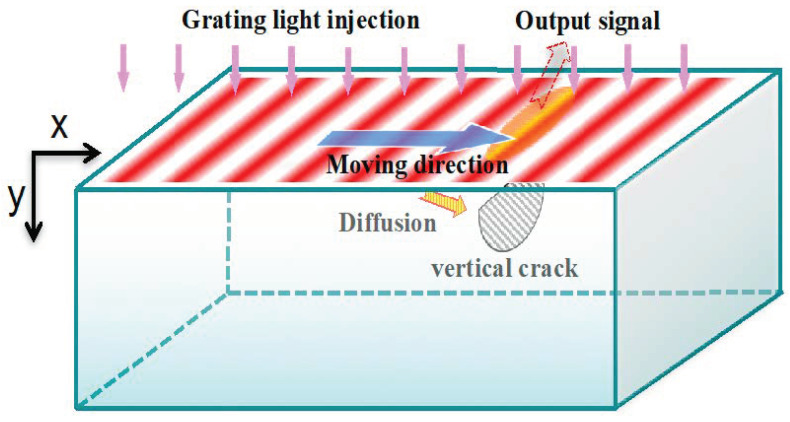
Illustration of infrared grating thermal wave scanning method. The red stripes represent the light gratings, they move from left to right along *x* direction. The light gratings generate heat flux at the surface and thus thermal waves form at the surface correspondingly. When the thermal waves meet cracks or defect, they will be reflected and will further propagate to the surface. By monitoring the temperature signals of the surface, the cracks can be detected.

In this method, a series of striped moving light is projected on the surface of the sample, i.e., a light grating with sinusoidal spatial intensity along the *x* direction, as shown in Figure 3. The grating wavelength l is the distance between two adjacent stripes of light. The incident light moves with a constant velocity along the *x* direction. After some time, the temperature distribution, due to the injected grating light, reaches steady state and it has as the following form:

(20) $$T(x,y,t)=T_{0}\sin\left(\frac{2\pi }{l}x-2\pi ft+\varphi_{0}\right)$$

where *T*_0_ is the amplitude of thermal waves at the surface; *f* is the temporal frequency of the input signal; l is the wavelength of the grating along the *x* direction at the surface; *φ*_0_ is the initial phase angle and is a constant. From Equation (1) we can see that this is a moving thermal wave along *x* direction with a velocity v=f×l.

Controlling the grating wavelength by adjusting the distance of the light gratings through the projector, or the temporal frequency *f* by changing the moving speed *v* of the light grating under a fixed grating wavelength *l*, the thermal wave propagates not only along the *y* direction, but also along the *x* direction. When it meets a crack, a reflecting thermal wave will propagate to the surface of the sample. The output signal of thermal waves can be detected by an infrared thermal camera. By adjusting the temporal frequency and moving velocity of the illuminating light in *x* direction, both the vertical cracks and horizontal cracks can be detected and located. The thermal wave response and thermal diffusivity are as followings:

(21) $$T(x,y,t)=T_{0}e^{-y/\eta }\left[\cos2\pi \left(\frac{y}{\kappa }+\frac{x}{l}-ft\right)+i\sin2\pi \left(\frac{y}{\kappa }+\frac{x}{l}-f\right)\right]$$

where η is the length scale of thermal wave diffusing into the solid, i.e., skin depth; κ is the wavelength of thermal waves in the vertical direction. These parameters satisfy the following relationship

(22) $$\frac{1}{\eta }=\sqrt{2}\pi \sqrt{\frac{1}{l^{2}}+\sqrt{\frac{1}{l^{4}}+\frac{f^{2}}{{\left(2\pi \alpha \right)}^{2}}}}$$

(23) $$\frac{1}{\kappa }=\frac{1}{\sqrt{2}}\sqrt{-\frac{1}{l^{2}}+\sqrt{\frac{1}{l^{4}}+\frac{f^{2}}{{\left(2\pi \alpha \right)}^{2}}}}$$

According to the existing research results and practical application, the infrared thermal wave imaging detection method is suitable to detect and monitor the development defects of fatigue damage such as crack, rust and debonding. Thermal wave detection is generally fast, has a large observation area, non-contact, and the result is intuitive, accurate, making it suitable for field application and online in-service detection. The advantages and disadvantages of the above specific methods are compared as shown in Table 3.

## 3. Infrared Image-Processing Technology

Infrared image is the most important evaluation basis of defect detection results. The purpose of infrared image processing is to filter out background interference and noise signals, enhance defect information and improve defect recognition, so as to extract more accurate and rich features from thermal image.

### 3.1. Analysis of Infrared Thermography Testing Factors

For the qualitative and quantitative detection of defects, there are many factors affecting the accuracy, and they can be grouped into the following categories: the influence of the infrared thermal imager system, heating mode, experimental technology, the influence of temperature data acquisition and processing [79,80,81,82,83,84,85,86,87,88,89,90,91,92,93,94,95,96,97,98,99,100,101]. The analysis of these factors can effectively improve the ability of defect detection, and it is also the key to achieve quantitative identification.

(1) Influence of the infrared thermal imager system

Temperature resolution, spatial resolution, and frame frequency are the most important performance parameters of a thermal imager. Resolving temperatures is the most critical index/factor for thermal wave detection. At present, the temperature resolution of mainstream infrared thermal imagers has reached 0.01 °C, which basically meets the requirements of infrared non-destructive testing (NDT). The scanning speed cannot be less than 25 frames/s; otherwise, the corresponding speed is too slow, and the error is too large. As for the influence of system noise and the error of the thermal imaging system during detection, an image-processing algorithm in the later stages of detection usually compensates for these [97,98,99,100,101].

(2) Influence of the heat flow injection direction

In infrared detection, the injection direction of the heat flow through the defect of a tested object will directly affect the test result. The oblique injection of a heat source will lead to non-uniformity of heat flow and obscure the test result; therefore, this is not ideal. When the heat flow is injected laterally, the surface of the object is parallel to the heat flow; therefore, the object can be heated at one end and cooled at the other end to reach a constant temperature. This is called steady-state heat conduction, and it is suitable for checking the shape of cracks. If the heat source is injected vertically, unsteady-state heat conduction occurs; this method is suitable for the detection of defects such as a blowhole, slag inclusion, incomplete penetration, and adhesion. In the vertical injection of heat flow, single-side heating or double-side heating also have great influence on the sensitivity of the measurement. Single-side heating can detect the temperature difference of in the cooling process after the heat source is removed; thus, this is suitable for a test object with complex geometry and constant thermal conductivity. Double-sided heating can perform detection during the heating process, which has high sensitivity; thus, this is suitable for metal materials with both high and low thermal conductivity [102].

(3) Influence of environmental factors

The influence of environmental factors primarily includes: radiation and reflection, the material surface and environmental convection on the detection signal, the material surface and environmental radiation heat transfer [103,104,105,106]. The temperature difference between the surface of the object to be measured and the temperature difference recorded by the thermal imager can always ensure the accuracy of the results. The maximum temperature difference between the defect area and the non-defect area is usually small. The infrared radiation energy m detected by the infrared thermal imager consists of three parts:

(24) $$M=\varepsilon \sigma T^{4}+M_{\alpha }+M_{\alpha tm}$$

$M_{\alpha }$—Radiation energy reflected from environmental medium to material; $M_{\alpha tm}$—Radiant energy of atmospheric medium entering detector.

If the value of $M_{\alpha }+M_{\alpha tm}$ is not equal on each pixel, even if the temperature and emissivity of each point on the material are equal, there will also be errors on the thermal image, which will affect the detection accuracy. Therefore, some measures should be taken to reduce the measurement error of these two factors.

In view of the adverse factors, by studying the influence degree, references for the actual detection process can be provided, for example, the mask is used to block the detection object to reduce the huge noise caused by environmental radiation. In additional, the characteristics of materials and defects also have an impact on the test results. For different types of materials and defects, appropriate test methods should be selected.

(4) Influence of heat source excitation parameters

**Figure 4:**
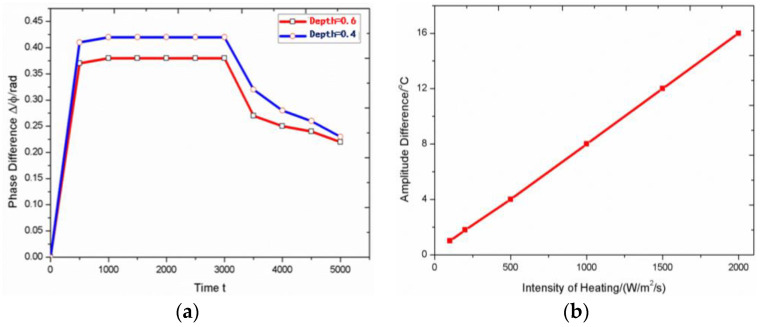
Influence of heating time and intensity on test results (**a**) time (**b**) intensity.

As mentioned above, different types of thermal excitation sources have different detection effects. The heating power and time of the thermal excitation source have great influence on the detection results. For infrared thermography testing, too short a heating time will lead to insufficient heat conduction, and too long a heating time will lead to uniform temperature distribution of the object, which will have adverse effects on the detection results. The increase of heating power can enlarge the temperature difference between defect and non-defect area. However, if the heating power is too high, it will destroy the measured object. Therefore, it is necessary to determine the appropriate heating time and heating power according to the material type and actual conditions, so as to ensure that the internal defects can be effectively detected. Take the grating infrared thermal wave testing method as example. First, when testing the influence of heating time, the phase difference is used as the judgement of detection result. When detecting defects (air) of different depth (0.4 mm and 0.6 mm) in steel specimens by numerical simulation [66], as shown in Figure 4a, the *x*-axis is the time (time characteristic point, related to the time step), the *y*-axis is the phase difference between defect area and non-defect area. The red curve is the detection result of 0.6mm deep defect, and the blue curve is the result of 0.4mm deep defect. Both of them show similar change rules: in the initial stage, with the increase of heating time, the heat conduction to the interior is sufficient, and the phase difference increases. When heated to a certain time, the internal heat conduction process of the object gradually tends to be stable, and the increase of heating time no longer has an impact on the detection effect. When the heating time continues to increase, the amplitude difference decreases and the detection effect becomes worse. This is because after a long time of heating, the temperature distribution in the interior of the object tends to be consistent. Long-time heating may even cause damage to the test piece. Then, one should test the effect of heating intensity, taking the amplitude difference between the defect area and the non-defect area as the judgment of the influence. The amplitude difference depends on many factors; here, we just study the influence of heating intensity on it. When detecting the 0.4mm deep defect, as shown in Figure 4b, the *x*-axis is heating intensity and the *y*-axis is amplitude difference. The amplitude difference is proportional to the heating intensity. The relationship shows that in a certain range of intensity, the detection effect can be improved by increasing the intensity of the excitation heat source. This is because the increase of intensity is conducive to reduce the influence of environment, noise and other factors on the detection results, and enhance the extraction of useful information.

For the testing method with the periodically modulated thermal excitation source, the frequency will also affect the detection results. The frequency is inversely proportional to the thermal diffusion length, and the appropriate frequency should be selected for different depth defect detection. Compared with low-frequency signal, high-frequency signal is more susceptible to noise and is not conducive to sampling. 

(5) Influence of material and defect parameters

Different detection objects will produce different detection results. For metal materials with high heat transfer coefficient, the detection time is only tens of milliseconds. However, for the composite with low heat transfer coefficient, the detection time is usually several seconds to tens of seconds. The heating time of the two is quite different. In addition, the surface reflectance of metal materials is higher than that of composite materials, and the original detection effect is not as good as that of composite materials. For defects with the same depth and size, the larger the difference of heat transfer coefficient between the defect and the material body, the easier to detect. In the process of heat conduction, the heating power decreases exponentially with the increase of depth, so the defect depth has a great influence on the detection results. Shallow defects are relatively easy to detect, and so are defects with large diameter and thickness.

Among the three factors of depth, diameter and thickness, depth has the greatest influence on the detection results, followed by thickness, and thickness the least.

This paper briefly introduces some main influencing factors. In the actual detection, it is necessary to reduce the adverse effects by reasonable planning and process the original thermal image.

### 3.2. Thermal Wave Image-Processing Method

For the original thermal image, the defect information can be difficult to observe because of environmental noise or unequal thermal excitation. In order to obtain a thermal image with a high SNR, a new post-processing method of the thermal image is used to obtain clear defect information. This image processing is generally divided into single frame image processing and continuous multi-frame image processing [107,108,109,110,111,112,113,114,115,116,117,118].

(1) Infrared image noise reduction technology

The infrared thermal imager mainly has the following kinds of noise: thermal noise, shot noise, 1/f noise, compound noise, fixed pattern noise, and more. The comprehensive effect of these noise sources makes the infrared image have poor contrast, a fuzzy image, an unclear edge and large noise, so it is very important to deal with the noise effectively.

The traditional infrared image denoising method mainly uses the linear filtering method, which has two methods: spatial domain and frequency domain. Spatial filtering often refers to the image itself, and directly processes image pixels, including mean filtering, median filtering, Gaussian filtering, and more. In frequency domain filtering, the signal is generally transformed to frequency domain, multiplied by the designed window function, and then inversely transformed to time domain, the window function is determined by the frequency component to be filtered. Frequency domain methods include high-pass filtering, low-pass filtering, homomorphic filtering, and more. In the case of low SNR, the SNR of the infrared image processed by the filter is not improved much, and the details of the image will be blurred. Wavelet transform has the ability of frequency localization analysis, which makes it widely used in the field of image noise reduction. It can effectively filter out the noise, retain the high-frequency information of the image, and obtain the best reconstruction of the original signal.

(2) Infrared image enhancement technology

In recent years, researchers have explored the enhancement of infrared image. There are three kinds of algorithms: the enhancement based on statistical histogram, the enhancement based on frequency change, and the enhancement based on digital details. The first two categories are easy to cause permanent loss of many details. Digital detail enhancement (DDE) is an advanced non-linear image-processing algorithm, which can compress large dynamic digital image and keep and enhance the details of image as much as possible. 

(3) Processing technology of infrared sequence thermogram

Due to the influence of noise of infrared thermal imager, uneven heating of excitation source and different absorption rate of material surface, the reliability of defect information in single frame infrared image is low, and some tiny defect information will even be submerged by noise. Therefore, infrared image processing is important, which is a key technology in the field of infrared thermal wave non-destructive testing. It is mainly used to eliminate the interference of adverse factors, improve the signal-to-noise ratio, and enhance the display of defects. There are several methods for image processing of the main infrared thermography testing techniques.

(1) Thermal Signal Reconstruction (TSR)

TSR can effectively eliminate the influence of the nonuniformity of the excitation source and the absorption nonuniformity of the material surface, and enhance the defect display. The specific method is to take the double logarithm of the image sequence in the cooling stage, then use the polynomial to fit the data, and then reconstruct the image sequence, and then obtain the first-order image and the second-order image by derivative of the time change, so as to reduce the influence of noise and image enhancement, as well as the absorption non-uniformity of the material surface. TSR was first proposed by Thermal Wave Imaging (TWI) company (Madison Heights, Michigan, USA) and has been put into practice.

(2) Lock-in Thermography

The image-processing methods mainly include Fourier transform, correlation function, four-point method and so on. Finally, the amplitude and phase images of thermal wave are obtained. Because the lock-in technology uses periodic thermal excitation, the signal-to-noise ratio of the thermal wave image can be increased by increasing the acquisition period to achieve high detection sensitivity.

Because the thermal response of the specimen surface is also a sinusoidal periodic change signal, four temperature measurement data ($S_{1},S_{2},S_{3},S_{4}$) with equal time interval at a certain point on the specimen surface in a modulation period are extracted. According to the properties of sine function, the expressions of amplitude and phase can be obtained as follows [107]:

(25) $$\phi (x_{l})=\arctan\left[\frac{S_{1}(x_{l})-S_{3}(x_{l})}{S_{2}(x_{l})-S_{4}(x_{l})}\right]$$

(26) $$A(x_{l})=\sqrt{{\left[S_{1}(x_{l})-S_{1}(x_{l})\right]}^{2}+{\left[S_{2}(x_{l})-S_{4}(x_{l})\right]}^{2}}$$

Research on data-processing algorithms based on unsteady heat transfer is important [75]. The four-point average algorithm is based on steady-state sinusoidal signal processing. However, the accuracy for a transient/brief/quick process is low; sometimes it is even impossible to obtain any signal. The excitation source of the infrared lock-in thermography technology is a heat source with sinusoidal modulation, a more accurate data processing method is provided: the four-parameter fitting method [108].

First, the sinusoidal signal is sampled as follows

(27) y(t)=Acos(ωt+φ)+C

where *A* denotes the ideal amplitude of the signal, *ω* denotes the ideal frequency of the signal, *φ* denotes the ideal phase of the signal, and *C* denotes the ideal Direct-Current(DC) offset of the signal; these are the four parameters needed to express any sinusoidal signal. Of course, this is not the only method of expression. We can use another four parameters to describe a sinusoidal signal

(28) $$y\left(t\right)=A_{1}\cos\left(\omega t\right)+A_{2}\sin\left(\omega t\right)+C$$

where cosine amplitude *A*_1_, sinusoidal amplitude *A*_2_, DC offset *C*, and signal frequency are also called the four parameters of the sinusoidal signal. These are equivalent to the amplitude, frequency, phase, and DC offset in the previously described four parameters. In addition, the data record sequence is known as time *t*_1_, *t*_2_…*t_n_* sampling of the *y*_1_, *y*_2_…*y_n_* sine wave amplitude.

Next, the estimated values of degree, cosine amplitude, and DC offset are defined as *A*_1_, *A*_2_, and *C*, respectively. The sum of squares of errors between the estimated values and the real values is then

(29) $$\varepsilon =\sum_{i=1}^{n}{\left[y_{i}-A_{1}\cos2\pi ft-A_{2}\sin2\pi ft-C\right]}^{2}$$

where *n* is the number of samples. If

(30) $$D=\left[\begin{array}{l} \begin{array}{ccc} \cos2\pi ft_{1} & \sin2\pi ft_{1} & 1\\ \cos2\pi ft_{2} & \sin2\pi ft_{2} & 1\\ \vdots & \vdots & \vdots \end{array}\\ \begin{array}{ccc} \cos2\pi ft_{n} & \sin2\pi ft_{n} & 1 \end{array} \end{array}\right]Y=\left[\begin{array}{c} y(t_{1})\\ y(t_{2})\\ \vdots \\ y(t_{n}) \end{array}\right]X=\left[\begin{array}{c} A_{1}\\ A_{2}\\ C \end{array}\right]$$

the least-squares solution of these three parameters for the sine function is

(31) $$X={\left(D^{T}D\right)}^{-1}\cdot \left(D^{T}Y\right)$$

when ω is unknown, it is assumed that the extremum of ω exists and is unique in the range of [ω-ω/p, ω+ω/p] for sinusoidal waveform sequences with p periods; therefore, ω can be found by a one-dimensional search in the range of ω ± ω/p. Moreover, the least squares fitting at the extremum is the result of the four parameters least square sine wave fitting. Then, ω is calculated as follows:

(32) $$\omega =\max\left[\omega_{M},\omega_{T}\right]$$

(33) $$\omega_{L}=\omega_{0}-\Delta \omega_{\max}=v/m-v/n$$

(34) $$\omega_{R}=\omega_{0}+\Delta \omega_{\max}=v/m+v/n$$

(35) $$\omega_{M}=\omega_{L}+0.618\left(\omega_{R}-\omega_{L}\right)$$

(36) $$\omega_{T}=\omega_{R}-0.618\left(\omega_{R}-\omega_{L}\right)$$

From the above results, the amplitude and phase are then

(37) $$A=\sqrt{A_{1}^{2}+A_{2}^{2}}$$

(38) $$\varphi =\{\begin{array}{c} \arctan\frac{-A_{2}}{A_{1}};A_{1}\geq 0\\ \arctan\frac{-A_{2}}{A_{1}}+\pi ;A_{1}<0 \end{array}$$

(3) Pulsed Phase Technology (PPT)

Pulsed phase technology is a relatively new active infrared non-destructive testing technology, which combines the previously discussed pulse thermal wave measurement technology and modulated thermal wave measurement technology [66]. It was first proposed by Professor Xaviar Maldague of Laval University in Canada in 1996 [67]. In modulated thermal wave detection, there is only one thermal excitation frequency per test; in contrast, there are many different frequency components in the pulse thermal wave measurement technology. Pulsed phase thermal wave detection can extract signals with different frequencies from the thermal pulse excitation through Fourier transform spectral analysis; this involves two technologies [68].

**Figure 5:**
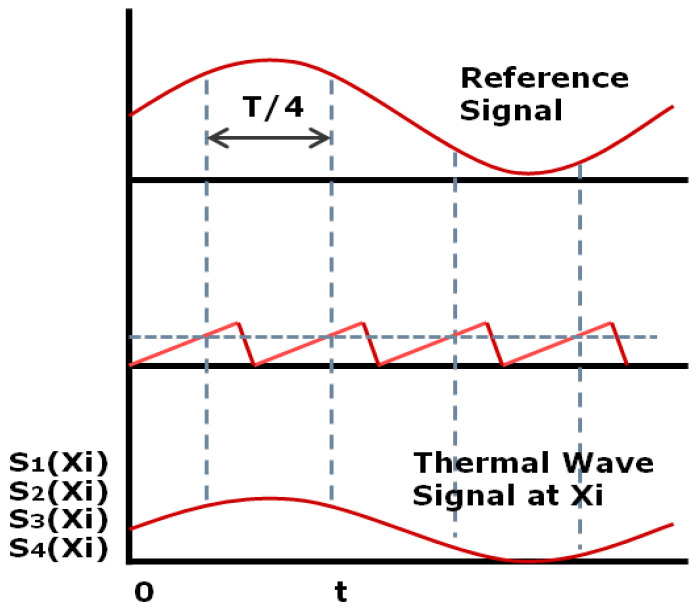
Principle of four-point average algorithm.

The pulse signal has a spectrum ranging from 0∼∞. The true thermal pulse with a limited width and amplitude is different from the ideal pulse δ. Therefore, the actual pulse excitation contains a finite number of frequencies, amplitudes, and phases of thermal excitation. The spectrum analysis of a square wave pulse as shown in Figure 5 indicates that the frequency distribution range is limited, and the amplitudes of different frequency components are different. The longer the delay time of the pulse, the narrower the spectrum will be; then, the energy will mainly concentrate on the low-frequency band. We also know that the lower the frequency of the excitation signal, the deeper the penetration depth will be. Furthermore, in pulse radiation measurement technology, more energy needs to be gathered in the low frequency region to enhance the ability to detect objects at greater depths [69].

With this, the vectors of each pixel in the thermal image sequence are transformed by one-dimensional discrete Fourier analysis. The real component Re*_n_* and imaginary component Im*_n_* are calculated to obtain the amplitude *A_n_* and phase*Φ_n_* by [70].

(39) Fn=∑k=0N−1T(kΔt)e−i2πnk/N=Ren+iImn

(40) An=Ren+Imn

(41) φn=arctanImnRen

Then, the surface temperature distribution of a medium with finite thickness d is heated by pulse heating using the formula

(42) $$T_{s}(0,t)=\frac{C}{\sqrt{4\pi at}}\left[1+e^{-{\left(2d\right)}^{2}/\left(4at\right)}\right]$$

Taking the natural logarithms on both sides,

(43) $$\ln T_{s}=\ln C+\ln\left(1+2e^{-d^{2}/at}\right)-\frac{1}{2}\ln\left(4\pi at\right)$$

Because thermal wave testing is mainly used for detecting the defects of shallow surfaces, the defect depth d is generally very small. The thermal diffusivity a of most materials is also a very small value, and the detection time t is usually several seconds to tens of seconds, $d^{2}/at$ can be regarded as an infinitesimal quantity, and $\exp\left(-d^{2}/at\right)$ can be approximated to 1. Therefore, Equation (43) can be simplified to

(44) $$\ln T_{s}=-\frac{1}{2}\ln t+b$$

where *b* is a constant related to the thermal diffusivity of materials with A and C.

**Figure 6:**
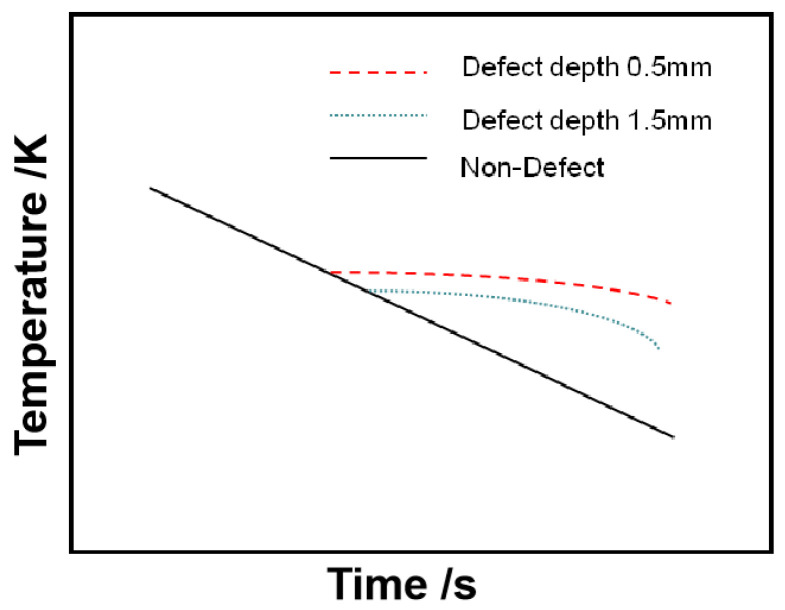
Surface temperature–time curves of test objects of different thickness (double logarithmic coordinates).

It can be seen from Equation (44) that when there is no defect in the material, the curve of logarithmic intensity and logarithmic time of the corresponding pixel points on the thermal wave image is ideally a straight line with a slope of −1/2. When there is a defect in the material, the thermal diffusivity *a* of the defective material is different from that of the non-defective material; the C value also varies. Then, the law of strength variation with time will no longer satisfy Equation (44), and the logarithmic curve of temperature–time will deviate from the reference line with the slope of −1/2 when the defect occurs. As shown in Figure 6, this happens at the time when the heat wave reaches the defect surface.

## 4. Quantitative Detection of Defects

The temperature distribution of the infrared pulsed thermography imaging testing is shown in Equation (7). When the thermal wave propagates to the defect at a depth d from the surface, it will be stopped and reflected. Then, the corresponding surface temperature of the defect area is found by

(45) $$T_{d}(0,t)=\frac{I_{0}}{\sqrt{\pi \rho ckt}}[1+2\exp(-\frac{d^{2}}{at})]$$

Finally, the surface temperature difference is obtained using the equation

(46) $$\Delta T=T_{d}(0,t)-T_{n}(0,t)=\frac{2I_{0}}{\sqrt{\pi \rho ckt}}\exp(-\frac{d^{2}}{at})$$

Therefore, after pulse heating, the defect depth can be determined based on time; this corresponds to the peak temperature difference in the equation

(47) $$t_{\max}=2d^{2}/a$$

Liu et al. [71] used the infrared lock-in method to detect defects, the heat wave transfer in the test piece was equivalent to an RC low-pass filter circuit. The defect depth is calculated by the phase difference between defect and no defect. The experimental results show that the measurement error is less than 5%. Jiang et al. [71] provided a method to detect the defect depth by using the time temperature double logarithm curve separation point when using infrared laser line scanning testing. By using grating infrared thermal wave scanning method, Zhang et al. proposed the grating infrared thermal wave scanning method and calculated the defect depth by substituting the corresponding frequency inflection point when the amplitude and phase tend to be stable and constant into the calculation formula of the wavelength in the vertical direction. The error of numerical simulation detection is within 2% [66].

## 5. Application of Infrared Thermography Testing

This technique was originally used in military applications but is widely implemented in power equipment detection, petrochemical pipeline leakage detection, smelting temperature and lining damage detection, aviation cementing material quality detection, landslide monitoring and forecasting, medical diagnosis, and other fields. Research and application of this technology for non-destructive testing have also been introduced for defect detection, identification of material thermophysical parameters, internal structural damage detection, building energy savings analysis, and house quality assessment [119,120,121,122,123,124,125,126,127,128,129].

(1) Detection of internal manufacturing defects in materials

Infrared thermography non-destructive testing can not only detect the internal defects of metal and non-metal materials, but it can also detect, identify, and evaluate damage in honeycomb materials, carbon fibers, and glass fiber multilayer composites; it is superior to other testing methods. This technology can also measure the thickness of materials, coatings, and sandwiches as well as recognize material and structure characteristics under the surface of the test object [92,93,94,95,96,97,98,99,100,101].

(2) Detection of thermophysical parameters of materials

Compared with other temperature measurement techniques, an infrared camera can measure the temperature of a large area quickly and accurately, and it has a wide temperature measurement range. Therefore, when it is necessary to accurately measure large temperature boundary conditions, an infrared camera has incomparable advantages. The study of the inverse heat conduction problem has many application prospects. In recent years, a great deal of research has been performed to develop identification of thermal physical parameters, boundary shape, boundary conditions and heat sources. In the research of the inverse heat transfer problem, infrared thermal imaging technology has been widely used to measure the temperature of the research object; this can solve the challenge of temperature boundary measurement conveniently and quickly. This method has been widely used in the research of inverse heat transfer problem.

(3) Structure internal damage and material strength detection

At present, structural damage research using infrared thermography includes concrete internal-damage detection, concrete fire-damage research, weld fatigue crack detection, carbon-fiber reinforced concrete internal crack detection, and more. Compared with conventional defect detection methods such as X-ray and ultrasound, infrared thermal imaging has the advantages of requiring no physical contact or miscible agent, a simple and convenient operation, and no radioactive hazards.

(4) Application in building energy efficiency test

As far as building energy-saving detection is concerned, Sweden began to use infrared thermography technique to detect building energy-saving and thermal insulation as early as 1966. Researchers in many countries, such as the United States and Germany, have also performed research work in this field. Because of the diversity of building facade forms and decorative materials, it is an important part of this research to develop special image analysis and processing software and to establish the basic database of emissivity of decorative materials inside and outside walls.

(5) Application in building leakage detection

The leakage of buildings includes the leakage caused by water supply pipeline and the rain water leakage caused by cracking of roof or exterior wall, because the moisture content of the leakage part is different from that of the normal part, and the temperature of the two parts is different in the process of heat conduction. Therefore, infrared thermography can be used to photograph the infrared thermal image of the wall in the abnormal humidity part, and the location of the leakage source can be found by comparing and analyzing with the direct observation results in the field.

(6) Application in electrical field

The test objects include transformer joint of air compressor in substation, conductor joint of distributor, transformer zero-wire joint, lighting joint of power supply plant, cable overload of tramway tunnel, temperature test of air switch joint and intermediate joint of high-voltage wire and cable, and more. Through the periodic temperature measurement of substations and transmission lines, a large number of potential safety hazards are eliminated, and unnecessary losses are effectively avoided, which provides an important guarantee for the safe operation of factories.

(7) Application in civil engineering

With the rapid development of infrared thermography, its application in civil engineering has also made great progress. This is especially true for building exterior wall decoration quality detection. By collecting the temperature field change of the external wall surface, the quality of the decorating project can be evaluated.

(8) Application in aerospace energy conversion detection

The turbine blade is the key component of energy conversion in aircraft. As the turbine blade rotates at high speed under the impact of hot gas, it not only bears periodic centrifugal force but also suffers from oxidation and corrosion. Therefore, the accurate and efficient detection of defects in the turbine blade is critical for preventing a catastrophic accident and improving the aircraft safety. Although the physical design of an internal hollow structure with a complex airway is used to improve the high-temperature resistance of the turbine blade [102], this still cannot meet the demand for thermal protection of the blade. Currently, the primary way to solve this problem is to use thermal barrier coating technology. The basic design idea is to deposit a coating on the surface of a superalloy to achieve thermal insulation and oxidation resistance. This takes advantage of superior ceramic material properties, such as high-temperature resistance, corrosion resistance, abrasion resistance, and thermal insulation. Y_2_O_3_ partially-stabilized ZrO_2_ (YSZ) is a common material in thermal barrier coating, which has excellent thermal and mechanical properties. However, thermal barrier coatings are sensitive to damage under the long-term, high temperature, high pressure, and high-speed rotating environment of a turbine; therefore, regular inspection of the thermal barrier coating is necessary to ensure that the blades can serve safely over time.

Under the above operating conditions, defect detection in the turbine blade primarily includes crack detection, cooling channel blockage detection, and damage detection of the thermal barrier coating. In [103], hot air is used as the excitation source to continuously excite normal and faulty blades; then, an infrared thermal imager is used to detect cracks by recording temperature changes on the surface of the blade. Shepard et al. [104] processed the time series of thermal images obtained by thermographic signal reconstruction (TSR) under pulsed thermal excitation; they reconstructed the temperature field inside the blade to determine whether the passage inside the blade was blocked or not. The research of Bison et al. [105] showed that pulse thermal imaging can not only detect the defects and thickness of the thermal barrier coating, but also further classify the defects using the characteristic parameters of thermal image signal transformation. Zhang et al. [66,85] proposed that the use of a grating heat source helps to detect tiny defects in the thermal barrier coating; this enables the thermal wave detection method to more effectively detect defects of very shallow depth.

(9) Stress detection

The infrared lock-in technique reflects the stress distribution of structural components by measuring the signal of temperature change caused by load excitation, and then quickly and accurately locates the local stress mutation position and stress concentration of structural components, so as to realize the rapid detection of structural components damage.

(10) Health care

When some physiological conditions of the human body change occur, the whole body or local heat balance is destroyed or affected, so the clinical manifestation is that the temperature of tissue increases or decreases. For example, in the diagnosis of breast cancer (the skin temperature of the mass is 1–3 degrees higher than the normal temperature), the temperature of human limbs is mainly determined by the state of blood circulation. In vascular disease, the blood circulation shows an abnormal temperature at the lesion site. The location and range of the lesion can be clearly displayed by infrared thermography.

## 6. Conclusions

Infrared thermography non-destructive testing has been gradually developed from laboratory research into a conventional testing technique which meets the needs of many engineering applications. It plays an indispensable role in fault diagnosis and extending the service life of products across many industries. Considering existing research results, this technique is progressing in accuracy, automation, intelligence, portability, and standardization. It has mainly developed in the following areas: (1) qualitative detection to quantitative detection, (2) the number of thermal imaging system parameters is increasing, which is improving the accuracy of test results, (3) information processing methods are becoming increasingly more accurate, thus producing smaller errors, (4) diversification, convenience, and accuracy of thermal loading is increasing, (5) adaptation to field testing requirements is improving, resulting in more portable systems, (6) current development of artificial intelligence is gradually enabling the capability of conducting the automatic identification of tests results.

## Figures and Tables

**Table 1:** Comparison of application and characteristics of various non-destructive testing techniques.

Technology	Test Object	Scope of Application	Advantage	Disadvantage
X-Ray testing	Internal defects	Casting, weldments, non-metallic products and composite materials, etc.	Not limited by material and geometry, and can keep permanent records. most sensitive to volumetric defects such as porosity, slag inclusion and incomplete penetration	not easy to find the crack perpendicular to the ray; not convenient to give the depth of the defect; strict requirements on installation and safety and is not suitable for on-site online detection; the detection cycle is long; the cost is high
Ultrasonic testing	Surface and internal defects	Forgings, weldments, glued joints and non-metallic materials	Sensitive to defects, quick results and convenient defect location	Difficult to detect small, thin and complex parts; coupling agent is needed; complex structure is difficult to detect; speed is slow; detection period is long
Magnetic particle testing	Surface and subsurface defects	Ferromagnetic material	higher sensitivity than ultrasonic or radiographic examination when testing surface defects of ferromagnetic materials; the operation is simple and the results are intuitive	Limited to ferromagnetic materials; difficult to measure the defect depth quantitatively
Penetrant testing	Surface opening defect	Various non loose materials	the equipment is simple, the operation is simple and the sensitivity is high. Display defects intuitively. especially suitable for the inspection of large work pieces and irregular parts as well as the maintenance and inspection of on-site parts	The process is complex; the test solution is volatile; only surface opening defects can be detected; surface porous materials cannot be detected
Eddy current testing	Surface and subsurface defects	Conductive material	The equipment is highly automated; not necessary to clean the surface of the test piece; time saving; no couplant required	Sensitive to the edge effect caused by part geometry and sudden change; easy to give false display
Infrared thermal wave imaging testing	measure the damage depth, material thickness and the thickness of various coatings and interlayer, as well as the identification of material and structural characteristics under the surface	Metallic and non-metallic materials	Fast; large detection area; the test results are intuitive. No contamination or contact of the test piece	More effective mathematical calculation model is needed to determine the depth of defects for structural parts with complex shape; the detection depth is not deep enough

**Table 2:** Comparison of pulse thermal excitation.

Method	Advantage	Disadvantage	Application
Flash	High power, high efficiency and high detection accuracy	Cumbersome volume, depth of detection	Metals, nonmetals and composites
Laser	High energy density, very uniform light intensity and high detection accuracy	Large volume, complex system and image time correction	Metals, nonmetals, composites and crackle
IR-Lamp	Wide wavelength range, stable power and portable	The depth of detection is low	Metals, nonmetals and composites
Hot air	Small size, easy to carry, cheap	The depth of detection is low and the energy is low	Less material for light absorption coefficient

**Table 3:** Comparison of advantages and disadvantages of various infrared thermography testing methods.

Method	Advantage	Disadvantage
Infrared Pulsed Thermography Testing	The heating mode is simple, fast detection speed and high efficiency	Not suitable for the detection of complex structural components, only for the detection of flat components. In addition, the uniformity of the heat source is very high and the detection depth is limited
Infrared Lock-in Thermography Testing	Large area for one-time detection; provide certain depth information; continuous thermal excitation modulation requires only a small amount of thermal load. Strong ability to suppress noise	For a specific defect depth, a specific frequency is required for detection, with low efficiency; the phenomenon of blind frequency, which is easy to be missed
Infrared Ultrasonic Thermography Testing	Strong penetration and high detection depth; high detection sensitivity and safe operation	Not easy to check the workpiece with complex shape, and the surface finish of the tested object is required to be high; couplant should be filled in the test piece
Infrared Laser Thermography Testing	High power density; high detection accuracy	The method of laser point heat source is limited by the small area of single detection and the longtime of detection process; the laser line scanning method requires higher signal sensitivity; the high-power laser may cause surface damage
Grating Infrared Thermal Wave Scanning Testing	Simultaneous detection of horizontal and vertical cracks; localizable detection; low requirement for sampling frequency of thermal imager	Lack of experimental verification; the existing heat sources are not satisfactory
